# Supplementary material for: Effects of combined exercise training for adults with resistant major depression: a pilot study from the TRACE-RMD project
Source: PeerJ. 2025 Nov 17;13:e20356. doi: 10.7717/peerj.20356 (PMC12633128; doi:10.7717/peerj.20356)
Supplement: Supplemental Information 1 [file peerj-13-20356-s001.pdf]

Versión 2.0.

Fecha: 27/04/2021

TITULO

PROGRAMA DE EJERCICIO FÍSICO EN PACIENTES CON TRASTORNO DEPRESIVO RESISTENTE A TRATAMIENTO: ESTUDIO TRACE-RMD.

Investigador/a Principal Nagore Iriarte Yoller

Servicio: Hospital Psiquiátrico de Álava (Red de Salud Mental de Araba, Osakidetza).

Como centro colaborador está la Facultad de Educación y Deporte- Sección CC. De la Actividad Física y del Deporte de la Universidad del País Vasco-Euskal Herriko Unibertsitatea (UPV/EHU).

1. ANTECEDENTES

1.1. Resumen

El presente estudio, observacional (con medidas de resultado psicopatológicas, bioquímicas y físicas), pretende determinar cambios a nivel afectivo y funcionales asociados al ejercicio físico (EF) en personas con depresión resistente al tratamiento. Las disfunciones en funcionalidad en estos pacientes son frecuentes y graves y su importancia contrasta con la ausencia de fármacos que alivien estos déficits. Los programas de aplicación de EF en estas personas, que inicialmente se desarrollaron como intervenciones orientadas a la mejora de la salud física en fases iniciales o en sintomatología leve, han demostrado que también pueden mejorar los síntomas de esta enfermedad en estadios más avanzados.

Se desea aplicar un programa de EF para estudiar cambios sintomáticos y a nivel físico, así como evaluar la mejora en la funcionalidad en una unidad de depresión resistente. Muestra: 20 pacientes con trastorno depresivo mayor resistente que realizarán un programa de EF. Antes y después de 12 semanas de intervención se valorarán las personas participantes con variables clínicas, físicas, fisiológicas, bioquímicas y de funcionalidad. Durante 3 meses se implementará el programa de EF 2 días por semana. Se aspira a encontrar una mejora en estado de ánimo y en funcionalidad, así como cambios en áreas físicas como la tensión arterial o índice de masa corporal que se consideran factores predictivos de la enfermedad cardiovascular.

1.2. Introducción. Antecedentes del tema: trastorno depresivo resistente y ejercicio físico

Depresión y depresión resistente-a tratamiento (DRT): necesidad no cubierta.
La presencia del trastorno depresivo mayor (TDM) en la sociedad actual es preocupantemente alta. Específicamente, la prevalencia-año del TDM en España es un 3,9% y prevalencia vida de 10,5%. La prevalencia-vida para hombres es de 6,29% y de 14,47% para mujeres en España según estudio ESEMED-España (Haro et al., 2006). Aunque no hay consenso internacional explícito, la literatura médica y la investigación define la depresión resistente a tratamiento (DRT) como todo TDM que no responde a dos ensayos de tratamiento antidepresivo en dosis adecuadas y durante una duración adecuada (Conway et al., 2017).

Las cifras de DRT son, también, llamativamente altas. Se estima que la proporción de pacientes que consiguen una remisión completa de síntomas tras tratamiento antidepresivo son del 50,4% (Wijeratne et al., 2008). Por el contrario, se cree que la proporción de personas con depresión mayor que no responden a farmacoterapia o psicoterapia es aproximadamente del 30% (Rush et al., 2006). En los casos en los que la depresión es tratada por un médico de atención primaria, el 32% responden parcialmente y el 45% no lo hacen en absoluto (Papakostas et al., 2010).

Cuando los pacientes con DRT se comparan con otras personas con TDM, este subgrupo muestra una peor productividad, una mayor comorbilidad y un mayor número de intentos de suicidio (Amital et al., 2008). Los estudios naturalísticos de tratamiento de DRT muestran que las tasas de respuesta al tratamiento son muy bajas: Dunner y cols. observaron que en una muestra de pacientes con DRT residentes en la comunidad, solo el 10%-año respondió a los tratamientos estándar para el TDM (Dunner et al., 2014).
El estudio más exhaustivo sobre resistencia a tratamiento antidepresivo realizado hasta la fecha (N=3 671) fue el STAR*D patrocinado por el National Instiute of Mental Health (NIMH) americano. Se trató de un estudio de alternativas secuenciales de tratamiento para aliviar los síntomas depresivos. Aunque con limitaciones, este estudio proporcionó una fotografía valiosa de lo que supone la DRT y la probabilidad estimada de éxito del tratamiento antidepresivo tras pruebas secuenciales de tratamiento. En el STAR*D los pacientes fueron sometidos a una serie de ensayos secuenciales de tratamiento antidepresivo y psicoterapéutico que poseyeran pruebas de eficacia. Las tasas de remisión aguda disminuyeron con cada nivel del STAR*D (nivel 1: 37%, nivel 2: 31%; nivel 3: 14% y nivel 4: 13%). El punto de inflexión en el que aparece una resistencia marcada fue el nivel 3 de tratamiento (tras el fallo de 2 ensayos en dosis y duración adecuadas). En este punto menos de 1 de cada 5 pacientes respondieron. En los ensayos subsecuentes (nivel 4) el fallo al tratamiento rozó el 90% (Rush et al., 2006).

Discapacidad y costos asociados con el trastorno depresivo mayor (TDM).
El TDM está asociado con un incremento de la carga de enfermedad por encima de la enfermedad cerebrovascular, cáncer y otras numerosas enfermedades médicas (WHO, 2004).
Es la primera causa de años vividos con discapacidad (DALY) en el mundo para jóvenes adultos (Lopez et al., 1998) y hay una predicción de que en 2030 será la segunda causa

de discapacidad en el mundo por detrás del HIV/SIDA. La carga económica de adultos con TDM en EEUU es 210,5 billones de dólares, 45% atribuidos a costes directos, 50% en costos de trabajo o empleo y un 5% relacionados con costos de suicidio.
En definitiva, los trastornos depresivos en general y TDM en particular está asociado con efectos adversos en el funcionamiento incluyendo la discapacidad. Dicha discapacidad es tan severa o incluso superior a la mayoría de las enfermedades médicas crónicas.
Además de ello, los trastornos depresivos suponen en conjunto el diagnóstico más comúnmente relacionado con suicidio. Su atención requiere el mismo grado de intervención urgente que el que procura el médico para el infarto de miocardio o el cerebral.

Trastorno depresivo y su resistencia a los tratamientos.
La primera etapa del tratamiento consiste en la introducción de antidepresivos de primera y segunda generación. El mecanismo de acción de estas familias de fármacos reside fundamentalmente en la intervención sobre las sinapsis cerebrales de manera que haya una mayor biodisponibilidad de aminas (serotonina, noradrenalina y dopamina). Cuando esta estrategia falla se suele recurrir a la combinación de antidepresivos con mecanismos de acción complementarios o a la adición de ansiolíticos benzodiacepínicos. El siguiente escalón ante el fallo de tratamiento consiste en la asociación con fármacos no antidepresivos (sales de litio, psicoestimulantes, tiroxina). Aunque el grado de evidencia para estas intervenciones es mucho menor y, en la práctica, rescatan un porcentaje reducido de casos con falta de respuesta (Guía de Práctica Clínica sobre el Manejo de la Depresión en el Adulto, 2014).
Adicionalmente se dispone de dos estrategias terapéuticas con alto grado de evidencia: en primer lugar, las intervenciones psicoterapéuticas centradas en la remisión de síntomas depresivos y la prevención de recaídas. En segundo lugar, la terapia electroconvulsiva (TEC) tanto para la fase aguda (remisión del episodio depresivo) como para la fase de mantenimiento (prevención de recaídas).
Desafortunadamente en el territorio histórico de Álava se reproducen las mismas cifras de resistencia a los tratamientos que han sido descritas en otras latitudes. Estos pacientes suelen permanecer con síntomas depresivos residuales de mayor o menor intensidad y con una gran afectación funcional en las esferas personal, familiar y laboral. Los costes indirectos de esta situación son, evidentemente, altísimos.

Otros recursos terapéuticos para la DRT disponibles en otros países.
En los EEUU la FDA (Food and Drug Administration) ha aprobado dos tratamientos no farmacológicos para el tratamiento de la DRT: la estimulación del nervio vago (Rush et al., 2005) y la estimulación magnética trasncreanal repetida (EMTr) (Dunner et al., 2014). Estos tratamientos no han sido aprobados aún por la EMA (European Medicines Agency) y tampoco se ofrecen en España en ninguno de los servicios de salud salvo puntualmente y con fines de investigación. Otros abordajes terapéuticos que son ofrecidos de manera “off-label” son fármacos opiáceos, óxido nitroso inhalado y ketamina administrada en perfusión IV (Bobo et al., 2016).
Recientemente la Unidad de Trastornos Afectivos de la Clínica Mayo en Rochester (Minnesota, USA) publicó su protocolo de tratamiento de la DRT con ketamina que ha

sido el modelo replicado por los ensayos de registro para la esketamina intranasal dirigidos por Janssen (Sanchez et al., 2017).

Avances en el tratamiento de la DRT a medio plazo.

En estos momentos se está viviendo una actividad importante en la investigación sobre nuevos tratamientos antidepresivos. La novedad de estos nuevos agentes reside en cuatro puntos fundamentales: 1) actúan sobre dianas moleculares completamente distintas a las que eran objeto de intervención por parte de los antidepresivos clásicos, 2) todos ellos median su efecto clínico por medio del aumento de la neuroplasticidad y la formación de nuevas sinapsis, fenómeno que sucede en cuestión de horas en paralelo a la aparición de sus rápidos efectos antidepresivos, 3) tienen un efecto de acción rápido, en cuestión de horas/días en comparación con las 3-6 semanas que requieren los antidepresivos actuales y 4) son efectivos en la DRT.

Las dianas moleculares sobre las que actúan son heterogéneas (glutamato, neuroesteroides/GABA, opioides, anticolinérgicos, antagonistas de la orexina). Todos ellos están en fase avanzada de desarrollo (II y III). La FDA y la EMA ya han aprobado la esketamina en administración intranasal de Janssen (Sanchez et al., 2018).

A los pacientes con DRT que por su complejidad son tratados en la unidad de depresión resistente se les ofrecerá participar en el estudio a través de su psiquiatra referente quienes verificarán que el paciente cumple los criterios de inclusión

El ejercicio físico y la salud en personas con depresión resistente.

El TDM es la principal causa de años vividos con discapacidad en todo el mundo representando el 11,9%. Se ha estimado que durante el año 2020 podría ser el segundo después de la cardiopatía isquémica en años de vida ajustados por discapacidad perdidos (Organización Mundial de la Salud, 2001). Ante este sombrío escenario, se han considerado diferentes estrategias no farmacológicas como posibles tratamientos complementarias al tratamiento farmacológico para ayudar a mejorar el pronóstico del TDM y las tasas de remisión, como el ejercicio físico (EF) y la terapia electroconvulsiva (Howland, 2010).

El sedentarismo es una característica muy común en la población con trastorno mental grave. Una de las causas para que esto se produzca es que tienen muy poca confianza en su capacidad de EF y no cuentan con el apoyo social necesario. Esta inactividad junto con otros factores de riesgo modificables como el consumo de tabaco, hipertensión arterial y sobrepeso/obesidad, además de los efectos secundarios de los tratamientos farmacológicos contribuye a la aparición de distintas enfermedades, así como disminución de la esperanza y calidad de vida (Mullor et al., 2017). El TDM puede actuar como causante o como repercusión de la inactividad física, es decir, el TDM puede ser la causa de que no se realice EF o puede aparecer por no realizarlo. Por tanto, existe una relación bidireccional (Almagro Valverde et al., 2014).

Desde hace varios años se reconoce que la realización de EF regular es cardioprotector, disminuyendo la incidencia de enfermedades cardiovasculares como hipertensión arterial, enfermedad de las arterias coronarias, diabetes tipo 2 y aterosclerosis (Powers et al., 2002). Además de ser cardioprotector, el EF ha mostrado una asociación positiva con el bienestar psicológico (Hassmen et al., 2000) y beneficios terapéuticos en personas

mayores con trastorno depresivo (Mather et al., 2002), síntomas de depresión en pacientes con Alzheimer. enfermedad (Teri et al., 2003) y MDD (Dimeo et al., 2001).

Aunque todavía existe cierta controversia sobre la intensidad y frecuencia del EF, y la duración del programa de EF, las recomendaciones eran la adopción de un programa de EF de intensidad moderada de al menos 30 minutos la mayoría de los días de la semana, durante 10-12 semanas (aan het Rot et al., 2009). En las últimas directrices de la Organización Mundial de la Salud sobre actividad física (AF) y hábitos sedentarios, para la mejora de la calidad de vida en adultos con TMD las recomendaciones generales no difieren de la población en general, es decir, deben acumular a lo largo de la semana un mínimo de entre 150 y 300 minutos de AF aeróbica moderada o bien un mínimo de entre 75 y 150 minutos de AF vigorosa, o bien una combinación equivale entre ambas con el fin de obtener beneficios notables en la salud. Así mismo, con el fin de lograr beneficios adicionales para la salud deben realizar dos días a la semana actividades de fortalecimiento muscular de intensidad moderada o más elevada (Bull et al., 2020).

Las posibles ventajas del EF como adyuvante terapéutico eficaz para el TDM son, en muchos sentidos, atractivas: el EF es un tratamiento económico que aporta muchos beneficios para la salud, mejora el bienestar general y puede llenar el lapso de 3 a 4 semanas que los antidepresivos requieren antes mostrando efectos terapéuticos (Fornaro and Giosue, 2010).

Sin embargo, antes de adoptar esta terapia claramente ventajosa, todavía se necesita más información sobre cómo las diferentes poblaciones clínicas, en diversos entornos, responderán a diferentes programas de EF. De hecho, en su metaanálisis, Lawlor y Hopker concluyeron que los efectos del EF sobre la depresión no se pudieron determinar debido a la falta de estudios de buena calidad, con controles adecuados, seguimiento adecuado y realizados en poblaciones clínicas (Lawlor and Hopker, 2001).

Mota-Pereira y colaboradores en un programa de EF de 12 semanas de caminatas de 30 a 45 minutos 5 veces por semana resultó en una mejora de todos los parámetros estudiados de depresión y funcionamiento: HAMD17, BDI, CGI-S y GAF. Además, los pacientes del grupo de EF mostraron una tasa de remisión del 26%, en comparación con el 0% del grupo de control (Mota-Pereira et al., 2011).

Aunque una prescripción de EF perfecta no existe, aún no está totalmente establecida la óptima para esta población. Para un diseño contemporáneo del EF es necesario basarse en el principio FITT (*i.e.,* frecuencia, intensidad, tipo y tiempo), pudiéndose manipular cada uno de ellos para conseguir los mejores efectos.

A pesar de haber una extensa información acerca del EF en personas con TDM (Mura et al., 2014), según la información actual existe una ausencia de documentación en relación con EF en personas con TDM resistente.

En resumen, existen evidencias de nuevas intervenciones terapéuticas para el tratamiento de la depresión resistente. Nuevas dianas farmacológicas que pretenden mejorar la evolución en la depresión su repercusión funcional augura que, en el futuro, los nuevos fármacos deberán ser combinados con otras estrategias terapéuticas incluida el ejercicio físico. Algunas evidencias sugieren que el EF aeróbico estaría asociado al aumento de la tasa de remisión en los pacientes que la reciben.

Por todo ello, **el objetivo de este proyecto** es integrar estos hallazgos en un estudio observacional que incluya la adición al tratamiento estándar según práctica habitual de la intervención de EF en pacientes con depresión resistente estudiando los cambios clínicos y funcionales. Igualmente se desea estudiar el impacto de esta estrategia terapéutica sobre los factores de riesgo metabólico que inciden en el acortamiento de la expectativa de vida que sufre este sector de la población.

2. BIBLIOGRAFÍA

Amital D, Fostick L, Silberman A, Beckman M, Spivak B. Serious life events among resistant and non-resistant MDD patients. J Affect Disord. 2008;110(3):260–4.

Almagro Valverde S, Dueñas Guzmán M, Tercedor Sánchez PA. Actividad física y depresión: revisión sistemática. Revista Internacional de Medicina y Ciencias de la Actividad Física y el Deporte. 2014;14:377–392.

Bobo WV et al. Ketamine for treatment-resistant unipolar and bipolar major depression: critical review snd implications for clinical practice. Depression Anxiety. 2016; 33: 698-710.

Bull FC, Al-Ansari SS, Biddle S, Borodulin K, Buman MP, Cardon G, et al. World Health Organization 2020 guidelines on physical activity and sedentary behaviour. Br J Sports Med. 2020;54:1451–1462.

Bradley, J., Howard, J., Wallace, E., Elborn, S., 1999. Validity of a modified shuttle test in adult cystic fibrosis. Thorax 54, 437-439.Conway CR, George MS, Sackeim HA. Toward an Evidence-Based, Operational Definition of Treatment-Resistant Depression When Enough Is Enough**.** *JAMA Psychiatry.* 2017;74(1):9-10.

Dimeo F, Bauer M, Varahram I, Proest G, Halter U. Benefits from aerobic exercise in patients with major depression: a pilot study. Br J Sports Med. 2001;35(2):114–7.

Dunner DL, Aaronson ST, Sackeim HA, Janicak PG, Carpenter LL, Boyadjis T, et al. A multisite, naturalistic, observational study of transcranial magnetic stimulation for patients with pharmacoresistant major depressive disorder: durability of benefit over a 1-year follow-up period: Durability of benefit over a 1-year follow-up period. J Clin Psychiatry. 2014;75(12):1394–401.

Fornaro M, Giosuè P. Current nosology of treatment resistant depression: a controversy resistant to revision. Clin Pract Epidemiol Ment Health. 2010;6(1):20–4.

Guía de Práctica Clínica sobre el Manejo de la Depresión en el Adulto. Guía de práctica clínica en el SNS. Ministerio de sanidad, servicios sociales e igualdad. 2014.

Haro JM, Palacína C, Vilagutb G, Cols. Prevalencia de los trastornos mentales y factores asociados: resultados del estudio ESEMeD-España Med Clin (Barc. Vol. 126. 2006. p. 445–51.

Hassmén P, Koivula N, Uutela A. Physical exercise and psychological well-being: a population study in Finland. Prev Med. 2000;30(1):17–25.

Howland RH. Therapeutic armamentarium for treating depression. Postgrad Med. 2010;122(4):66–93.

Lawlor DA, Hopker SW. The effectiveness of exercise as an intervention in the management of depression: systematic review and meta-regression analysis of randomised controlled trials. BMJ. 2001;322(7289):763–7.

Lopez AD. Murray CC The global burden of disease, 1990-2020 Nat Med. 1998;4(11):1241–1243.

Mather AS, Rodriguez C, Guthrie MF, McHarg AM, Reid IC, McMurdo MET. Effects of exercise on depressive symptoms in older adults with poorly responsive depressive disorder: randomised controlled trial. Br J Psychiatry. 2002;180:411–5.

Mota-Pereira J, Silverio J, Carvalho S, Ribeiro JC, Fonte D, Ramos J. Moderate exercise improves depression parameters in treatment-resistant patients with major depressive disorder. J Psychiatr Res. 2011;45(8):1005–11.

Mullor D, Gallego J, Cangas AJ, Aguilar-Parra JM, Valenzuela L, Mateu JM, et al. Efectividad de un programa de actividad física en personas con trastorno mental grave / Effectiveness of a Program of Physical Activity in People with Serious Mental Disorder. RIMCAFD [Internet]. 2017;67(2017). Disponible en: http://dx.doi.org/10.15366/rimcafd2017.67.008

Mura G, Moro MF, Patten SB, Carta MG. Exercise as an add-on strategy for the treatment of major depressive disorder: a systematic review. CNS Spectr. 2014;19(6):496–508.

Papakostas GI, Fava M. Pharacotherapy for depression and treatment-resistant depression. Hackensack NJ: World Scientific; 2010.

Powers SK, Lennon SL, Quindry J, Mehta JL. Exercise and cardioprotection. Curr Opin Cardiol. 2002;17(5):495–502.

Rot M, Collins KA, Fitterling HL. Physical exercise and depression. Mt Sinai J Med. 2009;76:204–214.

Rush AJ, Trivedi MH, Wisniewski SR, Nierenberg AA, Stewart JW, Warden D, et al. Acute and longer-term outcomes in depressed outpatients requiring one or several treatment steps: a STAR*D report. Am J Psychiatry. 2006;163(11):1905–17.

Rush AJ, Sackeim HA, Marangell LB, George MS, Brannan SK, Davis SM, et al. Effects of 12 months of vagus nerve stimulation in treatment-resistant depression: a naturalistic study. Biol Psychiatry. 2005;58(5):355–63.

Sánchez P. Novedades en psicofármacos. Presentación oral en las Jornadas Post-APA. Madrid y Barcelona. 2017.

Sánchez P. Novedades en psicofármacos. Presentación oral en las Jornadas Post-APA. Madrid y Barcelona. 2018.

Teri L, Gibbons LE, McCurry SM, Logsdon RG, Buchner DM, Barlow WE, et al. Exercise plus behavioral management in patients with Alzheimer disease: a randomized controlled trial: A randomized controlled trial. JAMA. 2003;290(15):2015–22.

Wijeratne C, Sachdev P. Treatment resistant depression: critique of current approaches. The Australian New Zealand J Psychiatry. 2008;42:751–762.

World Health Organization: promoting mental Health: concepts, emerging evidence, practice (summary report). 2004.

3. HIPÓTESIS

1.- La implementación del EF en personas con DRT mejorará los síntomas depresivos de la propia enfermedad medidos a través de la escala MADRS.

2.- La implementación del EF en personas con DRT mejora varios parámetros de salud física relacionados con el riesgo cardiovascular (composición corporal, capacidad cardiorrespiratoria, marcadores de metabolismo de la glucosa y lipídico y marcadores inflamatorios).

3.- La implementación de EF de personas con DRT mejora el resultado funcional de los pacientes evaluado mediante escala Sehaan de discapacidad.

4. OBJETIVOS

<u>Objetivo Principal:</u>

1. **Analizar cambios en los síntomas depresivos** valorados a través de la escala MADRS (Montgomery-Asberg Depressión Rating Scale).

<u>Objetivos Secundarios:</u>

2. Analizar **cambios en la severidad global** de la enfermedad a través de CGI-S (Impresión Clínica Global)

3. Analizar **los cambios en el resultado funcional y satisfacción** valorado a través de escala Sehaan de discapacidad o escalas autoevaluadas como QLDS Quality of life in Depression Scale o escalas de satisfacción con el tratamiento como Subject treatment satisfaction (TSQM-9)

4. **Mejorar en la salud física.** Para ello se tendrán en cuenta los parámetros que coinciden con los factores de riesgo cardiovascular y la reducción en la expectativa de vida que sufren estos pacientes: metabolismo lipídico y glucémico, composición corporal, presión arterial, capacidad cardiorrespiratoria, calidad de sueño y calidad de vida.

5. Estimular a los pacientes para la **continuación** de actividades de EF en otros recursos comunitarios.

5. MATERIAL Y METODOS

5.1 Diseño

Se trata de un diseño observacional, longitudinal, prospectivo, abierto y de medidas repetidas, en el que se aplicará un programa de EF, durante 12 semanas, con 2 momentos de evaluación al inicio y la finalización de la intervención.

5.2 Población de estudio

Los pacientes serán reclutados de forma consecutiva desde la Unidad de Depresión Resistente del Hospital de Psiquiátrico de Álava. Se tratará de una muestra de personas que viven en la comunidad, aunque se incluirán casos de pacientes hospitalizados. Estos últimos serían pacientes que de manera puntual podrían precisar ingreso hospitalario para control de los síntomas.

Inicialmente se plantea como un estudio piloto y por ello, se espera reclutar una muestra de unos 20 pacientes. Para el cálculo del tamaño muestral se realiza revisión bibliográfica y se propone el número de acuerdo con estudios previos (Mota-Pereira, J., 2011).

5.3 Criterios de inclusión:

A. Edad comprendida entre 18 y 75 años
B. Trastorno depresivo mayor, trastorno bipolar I o II.
C. Depresión resistente a tratamiento definida como resistencia a 2 o más antidepresivos
D. Consentimiento informado por escrito.

5.4 Criterios de exclusión:

A. Esquizofrenia u otras psicosis.
B. Presencia de riesgo suicida inminente

C. Enfermedades médicas inestables o inadecuadamente controladas.
D. Trastorno por uso de sustancias activo.
E. Comorbilidad con otras patologías psiquiátricas que constituyan el centro de atención principal de tratamiento.
F. Deterioro cognitivo anclado por la escala Montreal Cognitive Assesment <26/30.
G. Incapacidad para realizar EF secundaria a dificultades a nivel osteoarticular, cardiovascular o metabólico.

5.5 Criterios de retirada:

Los pacientes podrán retirarse del estudio en cualquier momento. La causa principal de la retirada se consignará en la página correspondiente del CRD. El investigador podrá retirar del estudio a los pacientes por los siguientes motivos, entre otros:

- La no asistencia a más de una semana seguida al programa de ejercicio físico.
- Retirada voluntaria.
- Lesión del participante que impide continuar con el tratamiento de ejercicio físico
- Embarazo durante la investigación.
- Pérdida de contacto durante el seguimiento.
- Decisión del investigador de que la continuación en el estudio no es lo mejor para el paciente (a analizar por especialista médico).

En el momento de abandonar el estudio, la razón principal para la retirada debe ser recogida y, si es posible, el paciente deberá ser reevaluado.

5.6 Intervención

A) **Evaluación** clínica realizada por parte del equipo investigador para confirmar el diagnóstico de trastorno depresivo resistente, así como evaluar situación clínica actual (MADRS, CGI), funcionalidad (escalas autoevaluadas por parte del paciente) y satisfacción con el tratamiento. Evaluación de los parámetros de salud física mediante pruebas de laboratorio y valoración de la condición física. Se realizarán en el Hospital Psiquiátrico de Álava las evaluaciones clínicas, de funcionalidad y de satisfacción de tratamiento, así como las pruebas de laboratorio y ECG. En las instalaciones de la UPV/EHU se realizarán las evaluaciones físicas (capacidad cardiorrespiratoria, mediciones físicas y cuestionarios).

B) **Intervención** propiamente dicha (programa de ejercicio físico). Será realizada en el Hospital Psiquiátrico de Álava. Los participantes realizaran una intervención mediante EF se ejercitarán de forma supervisada por especialistas en EF dos días por semana

durante 12 semanas y se les animará y asesorará para que incrementen su nivel de AF semanal para llegar a las recomendaciones generales. Todas las sesiones se iniciarán y finalizarán con mediciones de TA y la intensidad del ejercicio se controlará por pulsómetros de frecuencia cardiaca (FC) (Polar Electro, Kempele, Finland) y a través de la escala de Borg original (6-20). Todas las sesiones incluirán 10 min de calentamiento y 10 min de vuelta a la calma con estiramientos. La parte principal de la sesión consistirá en un circuito de 4 postas de 10 minutos de duración cada una: 1) ejercicio aeróbico en bicicleta, 2) ejercicios de fuerza-resistencia (cintas elásticas, propia masa corporal, mancuernas), 3) ejercicio aeróbico en bicicleta y, 4) ejercicios de fortalecimiento de la cintura pélvica. La intensidad se diseñará de forma individualizada en relación con la FC para desarrollar los rangos de intensidad ligera (valores de FC de reposo hasta valores FC del primer umbral ventilatorio) y moderada (valores de FC entre umbrales ventilatorios). Durante las sesiones en bicicleta la potencia y velocidad, se ajustarán para conseguir la FC marcada.

Protocolo en bicicleta:

Las personas participantes realizarán intervalos de 15 segundos en R2 intercalados con intervalos de 1 minuto en R1.

Protocolo de fuerza:

Se diseñarán 10 ejercicios de fuerza-resistencia incluyendo tanto el tren superior como el tren inferior abarcando los principales grupos musculares y coordinado con la respiración.

Protocolo de fortalecimiento cintura pélvica:

Se diseñarán 6 ejercicios incluyendo la musculatura anterior y posterior.

C) Al finalizar el periodo de la intervención, se llevará a cabo una **reevaluación** desde el punto de vista clínico, físico y funcional.

EVALUACIÓN ANTES (PRE) Y DESPUÉS (POST) DE LAS INTERVENCIÓN.

A- Exploración clínico-diagnóstica:

1. Obtención del consentimiento informado.
2. Historia clínica y pruebas complementarias: Exploraciones físicas y complementarias habituales en estos diagnósticos que se realizan de acuerdo con la práctica clínica habitual de la depresión (analíticas de orina y sangre con perfil básico, hepático y renal, marcadores inflamatorios e interleukinas) y determinación de tóxicos en orina.

B- Exploración clínico-psiquiátrica:

1. Cuestionario Montgomery-Asberg Depressión Rating Scale (MADRS), validación de las versiones en español de la Montgomery Anxiety Rating Scale para la evaluación de la depresión y de la ansiedad (Lobo y cols., 2002)
2. Impresión Clínica Global: CGI-S.

3. Hoja de registro del tratamiento psicofarmacológico, dosis y duración de los mismos.

C. Evaluación de funcionalidad:

1. Escala de discapacidad de Sheeham
2. QLDS Quality of life in Depression Scale

D. Evaluación de la satisfacción del tratamiento

1. Subject treatment satisfaction (TSQM-9)

E. Evaluación de salud física:

La **variable física principal** será la CCR (capacidad cardiorrespiratoria) medida a través del VO_{2pico} (volumen oxigeno pico).

Las **variables físicas secundarias** incluirán, la tensión arterial, la composición corporal, el perfil cardiometabólico, el nivel de actividad física, y la calidad de vida (**Tabla 1**).

Evaluación de la condición física.

Los participantes serán informados sobre las recomendaciones previas a estas pruebas con anterioridad. La condición física se valorará a través de una prueba objetiva cardiopulmonar (CPET).

La prueba CPET se desarrollará en un ergómetro bicicleta Lode Excalibur (Groningen, The Netherlands). El protocolo se iniciará con 40W con incrementos graduales de 10W cada minuto hasta el agotamiento con continuo ECG. La prueba se realizará con análisis de gases (Ergo CardMedi-soft S.S, Belgium Ref. USM001 V1.0) que será calibrado antes de cada prueba.

El VO_{2pico} se definirá como el valor más alto VO_2 obtenido al final de la prueba. Se considerará esfuerzo pico cuando al menos dos o más de los siguientes criterios se cumplan:

A. Fatiga del participante (>18 en la escala de BORG).
B. Intercambio respiratorio ≥1,1.
C. Consecución de >85% de la frecuencia cardiaca máxima predicha.
D. No incremento del VO_2 y/o FC con incremento de la carga de trabajo (Mezzani et al., 2012).

Al final de cada minuto se registrará la **sensación subjetiva al esfuerzo** a través de la escala de Borg.

La **TA** se medirá cada dos minutos durante toda la prueba.

Los umbrales ventilatorios (UV) (*i.e.,* UV1 y UV2) se valorarán según métodos estandarizados utilizando el V-slope y los equivalentes ventilatorios (EqV). El primer umbral ventilatorio (UV1) se identificará como el punto de transición en la pendiente de producción de dióxido de carbono (VCO_2) *vs* VO_2 de inferior a 1 a superior a 1, o también se identificará como el *nadir* en la relación del EqV del VO_2 *vs.* la carga de trabajo. El segundo umbral ventilatorio (UV2) se identificará como el *nadir* en la

relación del EqV/VCO_2 *vs* carga de trabajo (Mezzani et al., 2012). Después de finalizar la prueba, el participante permanecerá en reposo en la bicicleta cinco minutos más para registrar las variables en la recuperación. Se tendrán en cuenta todas las indicaciones absolutas y relativas para finalizar la prueba (Task Force of the Italian Working Group on Cardiac Rehabilitation and Prevention (Gruppo Italiano di Cardiologia Riabilitativa e Prevenzione, GICR) et al., 2006).

La identificación de los dos UV determinará los tres rangos o dominios para el diseño de la intensidad del ejercicio físico (R1, R2, R3):

A. **R1**-intensidad **ligera** a moderada con valores de FC por debajo el UV1.
B. **R2**-intensidad **moderada** a intensa con valores de FC entre UV1 y UV2.
C. **R3**-intensidad **intensa** a severa con valores de FC por encima del UV2 hasta la FC pico.

Cuando no sea posible identificar el UV2, los dominios de intensidad de ejercicio físico se definirán a través de la fórmula de Karvonen teniendo en cuenta porcentajes de FC de reserva; *i.e.,* intensidad moderada definida entre el 50-75% de la FC de reserva y la intensidad alta >76% al <95% de la FC de reserva (Mezzani et al., 2012).

Actividad física y comportamiento sedentario y hábitos de sueño.

La actividad física, el comportamiento sedentario y los hábitos de sueños serán valoradas de forma subjetiva (cuestionarios, International of Physical Activity Questionnaire-IPAQ (Craig et al., 2003).

La calidad de vida para la salud se valorará el cuestionario SF-36 (Laboratorio de Análisis del Rendimiento Deportivo de la Facultad de Ciencias de la Actividad Física y del Deporte de la UPV/EHU).

Tabla 1. Programa de pruebas de valoración en la Facultad de Educación y Deporte-Sección CC de la Actividad Física y del Deporte.

Sesión	Medición	Metodología
**DIA 1**	**MEDICIONES FÍSICAS**	
	Estatura (cm)	Estadiómetro
	Masa Corporal (kg)	Báscula
	Perímetros cintura y cadera (cm)	Cinta métrica no elástica
	Masa libre de grasa, masa grasa y agua corporal total	Impedancia bioeléctrica
	Electrocardiograma de reposo	Electrocardiograma de 12 derivaciones
	**ACTIVIDAD FÍSICA & COMPORTAMIENTO SEDENTARIO**	
	International Physical Activity Questionnaire	Cuestionario (Anexo 6)

	**CONDICIÓN FÍSICA**	
	Capacidad cardiorrespiratoria	Prueba de esfuerzo en bicicleta con medición continua ECG, tensión arterial, pulsioximetría
	**CALIDAD DE VIDA**	
	Cuestionario SF-36	Cuestionario

5.7 Variables

1. La evaluación de la depresión y la ansiedad se medirá a través del Cuestionario Montgomery-Asberg Depressión Rating Scale (MADRS), validación de las versiones en español de la Montgomery Anxiety Rating Scale (Lobo y cols., 2002)
2. La impresión Clínica Global: se medirá a través de la escala CGI-S.
3. Se realizará una hoja de registro donde constará el tratamiento psicofarmacológico, dosis y duración de los mismos.
4. Se medirá la funcionalidad del paciente a través de dos escalas: Escala de discapacidad de Sheehan y QLDS (Quality of life in Depression Scale).
5. Se evaluará la satisfacción del tratamiento a través de la escala TSQM-9 (Subject treatment satisfaction)
6. Se medirá la capacidad cardiorrespiratoria medida a través del VO_{2pico} (volumen oxigeno pico).
7. La actividad física, el comportamiento sedentario y los hábitos de sueños serán valoradas de forma subjetiva (cuestionarios, International of Physical Activity Questionnaire-IPAQ (Craig et al., 2003).
8. La calidad de vida para la salud se valorará el cuestionario SF-36 (Laboratorio de Análisis del Rendimiento Deportivo de la Facultad de Ciencias de la Actividad Física y del Deporte de la UPV/EHU).
9. Se evaluarán parámetros en el análisis de sangre que miden riesgo cardiovascular como metabolismo lipídico y glucémico, así como marcadores inflamatorios o interleukinas.

5.8 Análisis estadístico

Todos las variables clínicas y psicopatológicas expuestas serán recogidas en primer lugar en los CRD (cuadernillos de recogida de datos) asignados a cada uno de los pacientes y en segundo lugar se registrarán en una base de datos para su posterior análisis. Los análisis estadísticos de estas variables serán llevados a cabo mediante el paquete de datos estadístico SPSS versión 18.0. Para la comparación de variables repetidas se empleará un ANCOVA.

- **Cálculo del tamaño de la muestra**

Inicialmente se plantea como un estudio piloto y por ello, se espera reclutar una muestra de unos 20 pacientes. Para el cálculo del tamaño muestral se realiza revisión bibliográfica y se propone el número de acuerdo con estudios previos (Mota-Pereira, J., 2011)

5.8 Utilidad

Si comprobamos la hipótesis que se plantea podríamos utilizar el ejercicio físico como tratamiento en el abordaje integral de la depresión resistente. El ejercicio físico supervisado y dirigido por un profesional puede ser una alterativa terapéutica fácil de aplicar que puede estimular y ayudar en su recuperación. Se pretende de esta manera, ampliar intervenciones que se puedan ofrecer en la unidad de depresión resistente, conjunto con un tratamiento psicofarmacológico y psicoterapéutico. Además, es una intervención segura, con escasos efectos secundarios y aplicable a pacientes de todas las edades, ya que es posible individualizar.
La incorporación de un programa de ejercicio físico podría tener un beneficio añadido sobre problemas de salud importantes como la obesidad o enfermedades cardiovasculares. La prevención y promoción de la salud son objetivos necesarios a tener en cuenta en las intervenciones terapéuticas de las personas que sufren enfermedades mentales, y así consta en uno de los objetivos estratégicos que desde el departamento de Salud del País Vasco se persigue en torno a la atención del paciente crónico.
Finalmente potenciaremos la realización de ejercicio físico en grupo para fomentar áreas debilitadas en los pacientes con depresión resistente.

5.10 Sesgos y limitaciones del estudio.

Este tipo de estudio es muy vulnerable a los numerosos sesgos y, en consecuencia, no generará evidencias de alta calidad, limitación inherente a un estudio longitudinal. Además, se conoce que se deben tener en cuenta otras variables de confusión (tratamientos psicofarmacológicos, edad, sexo, comorbilidad…) que pueden influir en los cambios en los síntomas depresivos y resto de evaluaciones.
Sin embargo, desconocemos la adherencia de personas con trastorno depresivo resistente a un programa de ejercicio físico. A pesar de las numerosas llamadas a mejorar la salud física en este grupo de población, hasta ahora no se ha puesto en marcha un programa así en nuestro medio ni se ha evaluado su eficacia. Convendría valorar tras un tiempo, y habiendo realizado un seguimiento, la evolución de los pacientes y repetir una medición meses después.
Sería interesante poder realizar un ensayo clínico incluyendo grupo control y aleatorización dado que esperamos que se produzca un efecto sobre el curso natural de la enfermedad tras la realización de la intervención.

5.10 Aspectos Éticos

El estudio ha sido elaborado respetando los principios establecidos en la declaración de Helsinki (1964) última versión Fortaleza, Brasil 2013, en el Convenio del Consejo de Europa relativo a los derechos humanos y la biomedicina (1997), y en la normativa sobre investigación biomédica, protección de datos de carácter personal. Ley 14/2007 de Investigación Biomédica
El estudio cumple lo establecido en el REGLAMENTO (UE) 2016/679 DEL PARLAMENTO EUROPEO Y DEL CONSEJO de 27 de abril de 2016 relativo a la protección de las personas físicas en lo que respecta al tratamiento de datos personales y a la libre circulación de

estos datos. Los datos personales serán tratados por Osakidetza – Servicio Vasco de Salud. No se cederán datos a terceros, salvo obligación legal. El paciente será informado de que tiene derecho de acceso, rectificación, supresión de sus datos, y la limitación u oposición a su tratamiento. Todos los datos se tratarán según la Ley Orgánica 3/2018, de 5 de diciembre, de Protección de Datos Personales y garantía de los derechos digitales.

Además, podrá ampliar información en materia de protección de datos en la siguiente dirección web: http://www.osakidetza.euskadi.eus/protecciondatos.

El proyecto ha sido aprobado por el CEI (Comité Ético de Investigación) de OSI Araba.
