# Supplementary material for: Effects of combined exercise training for adults with resistant major depression: a pilot study from the TRACE-RMD project
Source: PeerJ. 2025 Nov 17;13:e20356. doi: 10.7717/peerj.20356 (PMC12633128; doi:10.7717/peerj.20356)
Supplement: Supplemental Information 3 [file peerj-13-20356-s003.docx]

PART: participant

Sex: 1 man; 2 women.

Age

Antidepresives

Antipsicotics stabilizadores

Diabetes: 1 yes; 0 no.

Smoking: 1 yes; 0 no.

BodyMass pre

Height (cm)

Body mass index pre

waist pre

hip pre

wais/hip pre

FFM% pre: fat free mass %

FWM% pre: fat water mas %

FM% pre: fat mass %

VO2peak.L/min.absolut.pre : Peak oxygen uptake absolut

VO2peak.ml/Kg.relativ.pre: Peak oxygen uptake relative

VCO2.L/min.pre: volume of carbon dioxide

Met.pre: metabolic equivalent of task

UV1.L/min.absolut.pre: Ventilatory thresholds 1 absolute

UV1.ml/kg/min.relativ.pre: Ventilatory thresholds 1 relative

UV2.L/min.absolut.pre: Ventilatory thresholds 2 absolute

UV2.ml/kg/min.relativ.pre: Ventilatory thresholds 2 relative

RERmedido.pre: Respiratory Exchange Ratio

Workload.Watt.rel.pr

Workload.Watt.abs.pre

HRrest.BICI: heart rate rest in bike

SBPrest.BICI: systolic blood pressure rest in bike

DBPrest.BICI: diastolic blood pressure rest in bike

HRpeak.BICI: heart rate peak in bike

HR.UV1 PRE: heart rate ventilatory thresholds 1 in bike

SBPpeak.BICI : systolic blood pressure peak in bike

DBPpeak.BICI: diastolic blood pressure peak in bike

BORGpeak.BICI: borg scale in bike peak

RERpeak.BICI t(min).BIKE: Respiratory Exchange Ratio peak in bike

km.BICI: distance in bike

Glucose

Cholesterol total

HDL

Aterogenic index

TG

LDL

Urea

Creatine

ALT

AST

Gamma-GT

Fosfatas alkaline

Proteins Totals

Albumin

Sodio: sodium

Potassium

Hb A1c %

Hb A1c mmol/mol

PCR: C reactive protein

Insulin

HOMA: Homeostatic model assessment

Testosterone

Interleukin 1 beta

Interleukin 10

Interleukin 2

Interleukin 6

PF PRE: Physical Functioning

RP PRE: Role-Physical

P PRE: Bodily Pain

GH PRE: General Health

VT PRE: Vitality

SF PRE: Social Functioning

RE PRE: Role-Emotional

MH PRE: Mental Health

PSC PRE: Physical Component Summary

MCS PRE: Mental Component Summary
